# Pathways to Permanence: Legal Status Transitions as a Key Mechanism in Skilled Migrant Selection and Settlement

**DOI:** 10.3389/fsoc.2019.00044

**Published:** 2019-05-22

**Authors:** Elizabeth M. Jacobs

**Affiliations:** Department of Sociology, University of Pennsylvania, Philadelphia, PA, United States

**Keywords:** skilled migration, H-1B visa, temporary migration regimes, visa stress, labor market assimilation, immigration law

## Abstract

Despite impassioned debates about immigration reform brewing in the U.S. government, researchers know remarkably little about how immigration policy shapes migration behavior. There is still much to learn about the composition of specific classes of admission, how long migrants stay in the United States, and the legal channels they follow to permanent residency or emigration. This paper takes a life course perspective on skilled migration to examine the micro-level processes and various pathways that lead to permanent settlement and emigration, and identifies legal status transitions as a key sorting mechanism in processes of immigrant selection. I find that migrants who successfully underwent a previous legal status transition were more likely to pursue permanent residence, but also saw a wider array of avenues to obtain a green card. The mismatch in some migrants' permanent settlement intentions and temporary legal status can lead to feelings of alienation and frustration in the immigration system and the U.S. labor market, driving some to emigrate or seek channels outside of the skilled migration program to procure a green card. The findings of this paper deepen our understanding of the processes that shape selection effects among immigrants and highlight the need for more robust and granular longitudinal data on legal status indicators.

## Introduction

American politics has been gridlocked by debates over immigration reform for the better part of the last 40 years. Bitter arguments about undocumented migration, asylum and refugee seekers, “The Wall,” and border enforcement essentially center around which immigrants the government wants to admit into the country. But despite the impassioned debates on Capitol Hill and at dinner tables across the country, there are still large empirical gaps in our understanding of who enters the country, and how. Researchers know remarkably little about how immigration policy shapes the composition of specific classes of admission, how long migrants stay in the United States, and the legal pathways they follow to permanent residency or emigration.

Taking a life-course perspective on skilled migration, this paper offers a micro-level examination into how skilled migrants navigate the U.S. immigration system and undergo legal status transitions. Drawing on 48 in-depth interviews with immigration lawyers and skilled migrants, I examine the conditions in which skilled migration becomes a pathway to permanent settlement, and what factors contribute to dropping out of the skilled migration system. I find that depending on the strength of their social ties to the United States and their level of familiarity with U.S. institutions, migrants holding the same legal status have vastly different migration histories and trajectories, leading to widely different approaches to pursuing a green card or emigrating. Where migrants with strong attachments to the United States often opt out of the skilled migration system and obtain green cards through family reunification channels because they do not see skilled migration as a viable option for permanent settlement, those with weaker ties are often more tolerant of the arduous process of applying for an employer-sponsored green card, as they consider it better relative to their options back home. A third group opts out of the immigration process entirely, choosing to return home or move abroad in pursuit of more enticing career prospects.

Examining migration trajectories and legal status transitions across the migrant life course reveals important selection effects and illuminates the processes underlying them. This paper makes two contributions to the research on immigration, one empirical and one theoretical. Empirically, the life course perspective offers a fresh way of understanding pathways to permanent settlement and emigration and reveals the processes and dynamics underlying the transition points that lead to drop out and selection effects. I broaden the focus of skilled migration beyond a singular migratory event to a process that unfolds over the life-course and that is shaped by other life course events and expectations (Massey and España, 1987). By taking a long view of the migration journey and comparing different migration pathways, I find important differences in who comes to the United States, how they arrive, and how they settle or emigrate. This paper pays special attention to the policy context in which skilled migration occurs, and identifies legal status transitions as a key sorting mechanism. On a theoretical level, the findings of this paper raise important questions about the ongoing capacity of the state in regulating and controlling immigration policy.

The implications of this paper will also be of interest to policymakers interested in understanding the migration pathways and settlement patterns of immigrants. How migrants navigate and make sense of the migration process is crucial in designing effective immigration policies and visa classifications. Specifically, the recruitment and retention of skilled workers has important implications for the growth of dynamic and burgeoning sectors of the U.S. economy as skilled migrants become an increasing share of the immigrant population in the United States.

## Skilled Migration and Legal Status Transitions Across the Life-Course

Institutions and policies set the stage for skilled migration. Through visa programs like the H-1B work visa and the F-1 student visa, companies and universities function as gatekeepers for entry to the United States. These institutions work together to create migrant pathways. For example, migrants sometimes see higher education as a gateway to obtaining access to a country's labor market (Bound et al., 2015; Kerr et al., 2016; Thomas and Inkpen, 2017).

This paper develops a previously overlooked approach to the study of skilled migration, broadening the focus of skilled migration beyond a singular migratory event to a process that unfolds over the life-course (Bailey and Mulder, 2017). Life-course research emphasizes the linkages and transitions between life events and centers in on how these events fit together to shape life trajectories (Elder, 1985, 1994). This perspective sensitizes researchers to event dependence—the linkages between the incidence of one event and another—and status dependence—the relationship between a status and the incidence of an event (Bailey and Mulder, 2017). This framework lends itself well to research on migration. For example, migration is not a singular event, nor is it linear: migration is a self-perpetuating social process that unfolds over the life course (Massey and España, 1987). Migration scholars have incorporated the life-course perspective to explain key dynamics of migration behavior, from how migration can precipitate or delay marriage, to how past migrations increase the likelihood for future migrations, to how migration itself can function as a key life course event (Massey, 1987; Hutchinson and McNall, 1994; McNall et al., 1994; Swartz et al., 2003; Parrado, 2004; Clark, 2013; Kõu and Bailey, 2014; Ramos and Martín-Palomino, 2015; Sabharwal and Varma, 2016; Parke and Elder, 2019). While data on migration histories is complex and limited, the New Immigrant Survey falls into this tradition as a nationally representative longitudinal study of new legal immigrants and their children (Jasso, 2003; Jasso et al., 2005).

Grounded in the concepts of event dependence and status dependence, this paper examines legal status transitions as a key mechanism in the process of immigrant sorting and selection. Where most research on immigrant selection effects focuses on health or immigrant earnings, I focus on legal status transitions as key life course events that have important implications for shaping the composition of different migrant pools and classes of admission (Jasso et al., 2004; Chiswick et al., 2005; Akresh and Frank, 2008; Borjas, 2014). Even in seemingly random legal status processes, like the H-1B visa lottery, structural and cultural factors play an important role in who ultimately obtains a visa. Large corporations dominate the H-1B visa pool, and the recruitment and hiring processes of skilled foreign workers is shaped by academic institutional affiliation, class background, country of origin, and gender (Kapur, 2010; Chakravorty et al., 2016; Hira, 2016). The degree of incorporation into U.S. society and access to institutional and informational resources to navigate the immigration system also play an important role in who persists in obtaining a visa and who drops out, either because they do not have sufficient resources or because they see more attractive options abroad.

Taking a life course perspective on skilled migration also provides a fresh look at return migration and permanent settlement trajectories. Any life course event could be the precipitating event to initiate migratory return. The return migration decision is complex and extends beyond rational choice theories proffered by economists—it is imbued with life course considerations for future career plans, family formation, and previous migration experience (Sabharwal and Varma, 2016)^1^ Because of paltry data collected on emigration, return migration is a widely understudied but crucial dynamic of international migration. The lack of information on selection into return migration clouds our understanding of immigrant incorporation and might erroneously deflate measures of incorporation as the “best of the best” migrants emigrate and return home (Castles and Miller, 2014; Sanders, 2018). Further, the motivations for return migration are not well-understood, and a better empirical grasp on these dynamics could help policymakers design more effective policies to retain foreign skilled workers. This paper aims to address this gap by illuminating the factors at play in the return migration decision.

## Skilled Migration and Legal Status

Research on labor migration to the United States has primarily focused on low-skilled seasonal labor programs like the Bracero program and the H-2 visa (Calavita, 2010; Hernández-León and Sandóval Hernández, 2017). This body of work demonstrates the stress, uncertainty and exploitation associated with contingent, low-wage, low-skill jobs, and the ways it intersects with tenuous legal statuses (Menjivar and Abrego, 2012). Employers take advantage of temporary and undocumented workers' precarious legal status as they provide poor working conditions, pay less than minimum wage, and offer little room for advancement in unskilled jobs in construction and the service sector (Simon and DeLey, 1984; Bean and Stevens, 2003; De Genova, 2004; Hall and Greenman, 2014).

Research connecting immigrant labor and legal status primarily focuses on low-skilled workers *without* “full” legal status—that is, a status that is not fully formalized or legalized, such as an undocumented status, or a liminal or provisional status like Temporary Protected Status (Menjívar, 2006). Undocumented status can block migrants from employment and social mobility and can delay integration into the labor market; it can create feelings of anxiety and dislocation, and has been found to undermine immigrant health, with ripple effects across entire families, including U.S. children of undocumented parents (Bean and Stevens, 2003; Menjívar, 2006; Arbona et al., 2010; Menjivar and Abrego, 2012; Bloch, 2013; Hall and Greenman, 2014; Dreby, 2015; Gonzales, 2016; Asad and Clair, 2018). This body of research has illuminated the central role that legal status, or lackthereof, plays in shaping an immigrant's life. However, this literature has largely overlooked how legal status and legal status transitions impact the lives of legal migrants. In essence, the research on legal status has emphasized the effects of not having legal status, and has not yet fully delved into the complex world of the experiences of those *with* legal status^2^. The limited research in this area suggests that legal status matters a great deal. For example, legal migrants experience specific forms of anxiety and stress associated with their legal status categories, what Jasso (2011) calls “visa stress,” and logistical concerns about providing legal status documentation can deter migrants from seeking healthcare (Hacker et al., 2011).

Further, the literature often conceptualizes legal status within a legal/undocumented binary (Massey et al., 2016; Sisk and Donato, 2016; Palter, 2017). Yet this simple contrast between legal and undocumented status does not recognize the range of legal statuses and the variation of experiences of different legal migrants (Asad, 2017). Studying legal immigrants as a homogenous category obscures important variation differences within legal admissions, which range from family visas, to work visas, to diversity visas. This variation in class of admission has considerable consequences: for example, legal migrants entering on a temporary visa earn less than legal migrants who enter on a permanent visa (Brownwell, 2010; Jasso, 2011; Mukhopadhyay and Oxborrow, 2012). In examining temporary legal migrants with high levels of human capital, this paper aims to expand the analytical scope of research on legal status and explore the key role of legal status transitions as a sorting mechanism across the life corse.

## The Neoliberal Role of the State in Immigration Policy

One of the central questions in the literature on globalization is whether the nation-state continues to be relevant in a globalizing world. Do the totalizing forces of globalization have the power to override national arrangements? Does the swell of transnational and supranational economic, political and social forces undermine national sovereignty? The rise of skilled migration, and the increasingly entangled capacity of state and company, reveals the complex and paradoxical relationship between the state and global forces.

Conventional theories of the state and migration emphasize the key role of immigration policy in nation building (Zolberg, 2006; Khoo and Hugo, 2008; FitzGerald and Cook-Martín, 2014; Czaika and de Haas, 2016). In constructing policies of admission, states differentiate between “desirable” and “undesirable” immigrants, revealing implicit biases about who constitutes an in-group member along lines of race, national origin, class and criminal background (Bean, 2016; Flores and Schachter, 2018; Pryce, 2018). A longstanding debate in the scholarship on immigration policy questions the extent to which governments can control the flow of immigrants. Where Zolberg (2006) suggests that countries can build a “nation by design,” essentially selecting the immigrants who make up the populace through immigration programs, Massey (2013) instead argues that immigration policies can produce unintended consequences and are in fact often a “fiasco.”

To some extent, there are challenges to the nation state from above and below. An increase in the power and legitimacy of privatized and denationalized authorities at the grassroots and supranational level challenges state sovereignty and marks a partial destabilizing of the nation state. Global dynamics, like international flows of capital, commodities, services, people, and information, are producing a “rupture in the mosaic of [state] regimes” and are expanding authority beyond state jurisdiction (Sassen, 2007, p. 222). At the same time, the state is surrendering some of its own authority through deregulation and supranational trade and legal agreements. In this way, the state hosts and enables denationalized agendas and processes.

Thus, a paradox: it is precisely because of the “highly developed functionality of the nation-state” that it has the capacity to produce the non-state capabilities that signal denationalization (Taylor, 1994, p. 416). As a result, we see the “destabilizing of some aspects of state power” and reducing some of its authority through processes of deregulation, privatization, and the construction of supranational entities, but it is also responsible for producing this “series of new legalities,” maintaining the state's central role (Sassen, 2007, p. 35). As Harvey notes, “while it would be erroneous to insist that traditional nation states have become irrelevant and powerless in relation to global capital, they certainly have become much more porous” (Harvey, 2006, p. 106).

Skilled migration policy in the United States is a key site to study the tension between state sovereignty and privatized non-state actors. Because skilled migration policy in the United States is predominantly employer-sponsored, the state and private companies work in tandem to regulate the entry and departure of skilled migrants. Visa programs like the H-1B and F-1 require institutional affiliation, which confers some bordering capacities to non-state actors like universities and companies. For example, because employment status and legal status are intertwined, companies have the capacity to terminate a migrant's legal status by terminating their employment. In another case, a student expelled from a university loses their student status and thus falls out of legal standing. As such, the bordering capacity traditionally reserved for the state has been partially transferred to the hands of private entities.

This paper thus illuminates some of the key dynamics of this debate. This interaction between global economic forces and state-level conditions offers a powerful analytic lens into how legal status and employment status can become intertwined, as in the case of the H-1B visa. By focusing on the relationship between immigration policy and private institutions like companies and universities, I aim to examine how shifting institutional arrangements are changing skilled immigration on a symbolic and functional level through changes in migration flows and the types of immigrants being recruited.

## Background and Context The H-1B Visa: The Case of Skilled Indian Migration

The expansion of skills-based visa programs has contributed to the significant growth of skilled migration to the United States in the past three decades. The United States is the top receiver of skilled migrants, with three times more skilled migrants than Canada and four times as many as the United Kingdom (Connor and Ruiz, 2019). About a third of immigrants to the United States are college educated, and educational attainment is trending upward: almost half of migrants who arrived in the last 5 years held a college degree.

The H-1B visa is the largest skilled work program in the United States and has had the greatest impact on the composition and recruitment of skilled foreign workers (Alba and Foner, 2015; Chakravorty et al., 2016). The H-1B is an employment-based temporary visa lasting 6 years in length, and is tied to a specific employer that applies for and sponsors the visa. As such, the legal status of H-1B workers is directly tied to their employer.

Each year, 65,000 H-1B visas are issued to private employers for workers holding a Bachelor's degree, with an additional 20,000 visas allocated to applicants holding advanced degrees. When the number of applications exceeds this threshold of 65,000, all petitions submitted before the cap is reached are entered into a lottery system. In recent years, demand has significantly exceeded the number of visas available—in 2016, 236,000 petitions were filed, and just over 30 percent of applications were approved (USCIS, 2016). The H-1B visa authorizes work in a number of specialty occupations, with the biggest concentration of H-1B visa migrants working in tech fields like software design, computer programming, and information technology. The median income of H-1B migrants in 2015 was $75,000 (USCIS, 2016).

Indian citizens make up the vast majority of H-1B migrants. Seventy-two percent of H-1B recipients in 2015 were Indian citizens, with Chinese nationals coming in second at eight percent (USCIS, 2016). The H-1B is the primary driver of the significant increase in Indian migration since the 1990's, as the U.S. technology sector has expanded (Chakravorty et al., 2016). Each year, tens of thousands of Indian migrants come to the United States on H-1B work visas and F-1 student visas, leading to a surge of Indian migrants in STEM fields and the IT sector (Chakravorty et al., 2016). These migrants, mostly male, come from specific sending regions within India, most commonly southern areas like Bangalore, Andhra Pradesh, Tamil Nadu. An additional 30,000 migrants entered each year on F-1 student visas, some of whom eventually transferred to an H-1B visa (Lowell, 2005).

### Pathways to the H-1B Visa: US Colleges vs. Employment Agencies

About a third of H-1B migrants move to the U.S. directly through work sponsorship on an H-1B visa (Hira, 2016). Others transition to the H-1B from other visas, such as student visas like the F-1 or J-1 visa (Chakravorty et al., 2016). It is not immediately clear using administrative data to determine whether an H-1B migrant intends to settle permanently. The H-1B visa is “dual intent”—the visa is temporary and “non-immigrant,” but there is a pathway to permanent residency through the EB visa program^3^,^4^. Jasso's (2009) analysis of the New Immigrant Survey suggests that work visa holders are less likely to express settlement intentions in the United States compared to other immigrants, yet other studies show that H-1B visa holders adjust to Legal Permanent Residence status at higher rates than F-1 visa holders (Lowell, 2005; Batalova, 2006)^5^. Further, evidence suggests that H-1B visa holders are more likely to seek LPR status through employer-based green cards, while migrants who originally arrived on an F-1 student visa often obtain green cards through other means like family reunification or marriage, depending on which pathway is most advantageous and efficient (Jasso et al., 2000; Jasso, 2009). The average wait times for LPR status is about 4 years, though the queue can reach up to 10 years or longer for citizens hailing from countries with more applications than the annual nationality quota can absorb (Jasso et al., 2010; Kandel, 2018).

A robust migration industry of subcontracting and outsourcing companies mediates the recruitment process between workers and employers. Indian emigration laws require potential migrants to register for emigration clearance before leaving the country and match with a recruitment agent. On the receiving end, migrants cannot obtain visas like the H-1B in the United States without employer sponsorship, emphasizing the key role of employers as brokers in obtaining a skilled work visa. Subcontracting firms like Tata Consultancy Services and Infosys are playing an increasingly large role in matching skilled workers to employers, adding another institutional actor to the process of skilled employer recruitment in the United States (Hira, 2016). Of the top 10 H-1B employers in 2017, five were high-tech employment services headquartered in India (USCIS, 2018). There is some debate as to whether outsourcing companies like Infosys and Tata Consultancy are “gaming” the system by flooding it with visa applications (Hira, 2016). Subcontracting companies rarely sponsor H-1B workers for green cards—in 2013, for example, Infosys sponsored only seven green card applications, while it sponsored 6,269 H-1B applications (Hira, 2016).

All skilled migrants arriving from India are positively selected in terms of education and class background relative to the total Indian population. By definition, skilled migrants hold a college degree, which makes them highly selected in a country where only eight percent of the total population is college-educated, and this inequality is based in large part on class differences (AISHE, 2018). Additional selection effects exist between international students and direct recruits, however. International students often come from more elite class backgrounds, having attended the most elite private high schools in India. Because international students rarely qualify for U.S. financial aid, most come from families that are able to pay out of pocket for tuition at a U.S. university, which can often cost more than $50,000 annually. The selection effects in terms of skill and “quality” are less clear: admissions rates at the most selected Indian Institutes of Technology are often lower than the top-ranked U.S. universities. In some cases, two percent of applicants who have passed a series of challenging entrance exams are accepted to Indian schools, making Harvard and Yale's six percent acceptance rates look promising in comparison (Najar, 2011). Some have noted a trend of American universities becoming “safety schools” for those applicants just shy of the mark.

Legal status transitions produce additional selection effects among international students as they transition from an F-1 student visa and H-1B visa. The transition onto the H-1B from a student visa might positively select for migrants in this pool who always planned to settle in the United States. Further, those who undergo this legal status transition might also have more information, social support and resources to successfully navigate the immigration system than those who drop out.

I expect the settlement intentions of H-1B visa holders in this study to be largely shaped by their entry point to the U.S.: whether they first came as an international student, or were directly recruited by a company to work in the U.S. These experiences are vastly different, and give U.S. degree holders three advantages: first, they become an H-1B migrant having already lived in the U.S. for at least 2 years as a student, which has exposed them to American culture and social networks. Second, their time in the U.S. has also given them experience navigating the U.S. immigration system: having previously held an F-1 or J-1 student visa, they have a slight edge in understanding how to navigate the complexities of obtaining a visa. Third, they come from more elite class backgrounds. Those who enter the U.S. visa system directly from India may well have different experiences. They have the advantage of not having to look for a job, since the recruiting agency has already navigated this process. But they face higher levels of adjustment once they have arrived, and they are likely to be particularly dependent on their employers.

## Data and Methods

This article is based on data collected from 48 semi-structured in-depth interviews: 33 with H-1B migrants from India, five with would-be H-1B migrants who were not able to obtain a visa, and 10 with immigration lawyers. All H-1B respondents in the sample work for private companies and held an H-1B visa in the past year. I supplement these data with interviews with immigration lawyers, who provided a more holistic view of the process and the common stumbling blocks that prevent some potential H-1B migrants from getting a visa. This study was carried out in accordance with the recommendations of the Institutional Review Board and the protocol was approved by the University of Pennsylvania IRB review board. Written informed consent was obtained by all interview participants.

Interview questions covered a range of topics related to the job search process, employment satisfaction, migrants' initial expectations and understandings of the U.S. immigration system, settlement intentions, and respondents' sense of belonging in the United States. This interview data offers an in-depth look at the ways skilled migrants make sense of and navigate the U.S. immigration system. The interview sample is not representative of the entire population of H-1B migrants in the United States, and it is not intended to generate systematic or generalizable observations about migration outcomes. Rather, the findings of this paper aim to illuminate new insights into the process of skilled migration, and to highlight meaningful dynamics that future researchers can investigate with representative samples. This paper sensitizes us to the factors at play at each transition point in the migration journey, which can help us better understand why some migrants stay and some migrants leave, and the potential implications this might have for selection effects.

Subjects were recruited through LinkedIn groups for H-1B migrants, as well as through alumni networks from public and private colleges and universities. From these diverse starting points, I supplemented my recruitment through snowball sampling. While this sample is limited in size and was not randomly selected, there were many distinct points of entry and thus initial respondents had a limited impact on the selection of subsequent interviewees. About three quarters of potential respondents contacted for this study agreed to participate; others said they were not interested in being interviewed or did not respond. Interviews, which were conducted between the spring and early fall of 2016, were conducted in person, and by Skype or telephone when the distance was too great to travel (for example, respondents living in India were contacted via Skype). Interviews lasted between 35 min and an hour and 42 min, with an average length of 53 min. Some respondents were contacted for follow-up interviews to clarify and further develop certain points. Interviews were transcribed and coded using NVivo. Names and identifying details have been modified to protect the identities of respondents.

Of the 33 H-1B migrants interviewed, 13 respondents in the sample were women and 20 were men. They ranged in age from 23 to 32 years old, with a median age of 28. Respondents varied in their time living in the United States, ranging from 3 to 8 years, with a median of 5 years living in the U.S. The median years on an H-1B visa was three, with a range of one to eight. The H-1B is a 3-year visa, with an option to renew it for an additional 3 years for a total of six, but once an applicant begins the green card process, their H-1B visa can be extended while the paperwork is being processed. The majority of respondents (N = 28) are currently living in the U.S.; five held H-1B visas but returned to India before their visa expired to pursue employment opportunities back in India.

Twelve respondents were foreign educated, with degrees from Indian universities; 21 respondents earned a degree in the United States. Among these 21 respondents, 11 earned their undergraduate degree in the U.S.; 10 went to college in India and earned a Master's degree in the United States. I define any respondent who earned any degree in the United States (Bachelor's, Master's, or doctorate) as U.S.-educated, because of the weight U.S. credentials carry in the labor market^6^. As previously noted, place of education and class of admission are closely related categories, but not perfectly correlated. All of the respondents in this study who were directly recruited to the U.S. earned their Bachelor's degrees in India and had 2–5 years of work experience in India before transferring to the United States. In this sample, direct recruits tended to be older, because they already had a few years of work experience before moving to the United States.

## Findings: Migrant Pathways and Uncertain Futures

This section is structured in three parts, to examine three key points in the process of skilled migration. First, I examine the factors leading to the initiation of migration, then I focus on the experience of being a skilled migrant, and finally explore variation in the settlement vs. emigration process.

### Initiation of Migration

The initial motivations for migrating to the United States varied widely between international students and direct H-1B recruits. International students migrated to the United States with goals of developing specialized skills and gaining specific credentials, and saw a student visa as a pathway to permanent residency in the United States. In contrast, direct recruits were often assigned a position in the United States seemingly at random, and often accepted the post with little intention or desire to settle permanently.

International students saw their time in the United States as an opportunity to develop skills and earn degrees from American universities. Sathvik, who is in his late 20's and earned his Master's degree in engineering at a public university on the East Coast, moved to the U.S. because of the specific educational opportunities it presented him. “I wanted to work in the field of computer architecture, and the universities in India don't have very good programs for getting a master's in that field,” he said. “Getting that degree and gaining those skills” were his primary motivations for migrating. Ridhi, who is in her late 20's and works for a tech company in Austin, decided to get her Master's degree in the United States because she was attracted to the career opportunities in her specific field. “I came to the U.S. for my Master's because of my interests in machine learning and data science,” she said. “There's not a lot of people doing interesting innovative work in that area in India, so it made more sense to get a background in that and gain work experience [here].” Both Sathvik and Ridhi eventually transitioned from an F-1 visa to an H-1B.

International students saw their studies in the United States as a gateway to permanent immigration. “Growing up, I always wanted to live in the United States. So when it came time to decide where to go to college, there was no question… this was my chance to move to America,” said Nisha, who studied business at a university on the East Coast and now works for a technology start up company in New York. “I knew that I was moving away at 18, and I would probably never come back.” Karan echoed similar expectations upon deciding to earn his Bachelor's degree at a university in California. “I remember buying my plane ticket for the beginning of college orientation. It was a one way ticket. And I remember thinking, this is it, this is my new home.”

Direct recruits, in contrast, saw their migration as a temporary work post. Most had never indicated any interest or intention in moving to the United States before they were approached by their employer with a project overseas. “I didn't particularly want to move [to the U.S.], to be honest,” said Roshan, who worked for a subcontracting company in Bangalore for 4 years before moving to the U.S. on an H-1B visa. “I wasn't so sure because I had no goals of moving to the U.S., relocating away from my family and my friends, nothing of that sort. But my boss called me in and told me they had a project for me in Seattle, so I said I would give it a shot.”

Respondents who were directly recruited saw migration as an opportunity to develop new skills by moving to the United States on an H-1B. “The position they were offering me in the U.S. was actually a promotion from what I was doing before, so I figured I would be able to learn a lot by moving over here,” said Myan, who worked for a software engineering company in India before transitioning to their U.S. team.

### Navigating the H-1B System and the U.S. Labor Market

For respondents who initially migrated on an F-1 visa, the legal status transition from a student visa to an H-1B highlighted how their permanent settlement intentions conflicted with the randomized and temporary design of the H-1B program. They described the job search process as “stressful,” “frustrating,” and “limiting,” due to the need to obtain an employer-sponsored visa, and the lack of guarantee that they would obtain a visa even if they did find an employer match. As Ridhi described her job search experience, she highlighted the constraints she faced, due to the employer-based nature of the visa. “I can't just go work for whatever company I want,” said Ridhi. “I *have* to work for a company that will sponsor an H-1B.”

Respondents often got far into the hiring process, only to be turned away once the conversation turned to work sponsorship. Jai, who got his Master's degree in engineering at a private university on the East Coast, spent the entirety of his 2-year Master's program searching for an employer that would sponsor him.

[The job search] was a very frustrating and enlightening experience. I realized the opportunities for an international student were quite limited. Almost every interview I had, about 80 percent of the interviews I had, stopped once I told them I needed to get an H-1B sponsorship. They were interested in me as a candidate but the visa thing was a hurdle…There's always going to be some opportunities that are going to be closed off to me just because I'm a different nationality.

Mira, a 27-year-old manager at a design company who holds a U.S. degree, echoed Jai's frustrations.

I got a lot of interviews, and got to the last round of the interview – they were ready to hire me before they realized that they couldn't because of the H-1B. They didn't sponsor foreigners. It was really frustrating – a waste of my time, going through all of those interviews for nothing.

The constraints of H-1B sponsorship shaped migrants' behavior in the job search. Perceptions of limited sponsorship stopped some respondents from applying to certain jobs. Shivani, a 28-year-old computer programmer who earned her Master's on the East Coast before moving to Seattle, limited her job application pool to jobs that she thought would sponsor her.

Some jobs require permanent residency or citizenship. Even really niche jobs, really happy jobs. It's not always clear what the requirements are, but if I thought there were citizenship requirements, I felt like I can't even apply to those jobs, so I didn't. I thought about it every once in a while but not too much. They have their reasons but it would be nice if lower level positions were more open, and there was more I could have applied for.

Other respondents perceived certain sectors as more likely to sponsor H-1B's, and recalibrated their career aspirations to obtain a visa. Rupi, 29, works at a software company in Seattle and earned her Bachelor's degree on the East Coast, where she was the editor of her college newspaper. She wanted to pursue a career in journalism, but “newspapers don't sponsor people for H-1B's,” she said. “So I…followed everyone else into tech.” Jai also avoided certain sectors of the economy, perceiving them as closed off to foreign workers, even though he had specific skills in that area. He “always wanted to go into mechanical engineering,” and earned his Master's degree in the field. But he was unable to find a job because “nobody would sponsor [him]. So [he] had to give up and get a job in tech instead.”

These issues did not apply to those who came directly from India with an H-1B visa in hand. In fact, it was the specific skills match that brought them to the United States. “They picked me for the original project here because I was the best guy in the company for it,” said Nishant, who studied computer science in Bangalore before moving to New York. “I'd been working on systems analytics since I started [at the company], and that's what I studied in college too. So it made sense that they wanted me to come over here to work on it.”

For students seeking work authorization, the uncertainty and frustration of the job search did not end once a company hires an H-1B worker and agrees to sponsor their visa. The employer then needs to obtain the H-1B through the random lottery process, which currently has about a one-in-three selection rate. By definition, every respondent in this sample “won” the H-1B lottery^7^. But the randomness and unpredictability of the lottery system was a common source of anxiety. “You sense a lack of control of your own destiny,” said Jai. “You're just waiting and waiting.”

Respondents who transitioned from a student visa to a work visa described the lottery process as acutely stressful. Many worried about falling out of status and needing to leave the country if their application was not selected. “I was really unsure what was going to happen while I was waiting for the H-1B,” said Aditi, who studied finance in the United States. “I wasn't sure if my life was going to go on here or if I'd have to move home. When my H-1B got approved I let out a huge sigh of relief. It's a huge weight off your shoulders. You just never know if something's going to go wrong.”

Some respondents were not selected in the randomized lottery process and decided to move home, rather than pursue legal status through other channels. For Sameer, the complexities and uncertainty of the migration system pushed him away. “I gave up on living in America after my H-1B was denied,” said Sameer, who went to college in California before moving back to Mumbai. “Too much of a headache, too much paperwork to stay. I did everything right, I went to a good school, I got the right job, and then I was just randomly rejected. It's better to be home, anyway.”

Direct recruits went through the same lottery process, but did not express the same feelings of anxiety and stress. Applying from their country of origin, they only commit to moving to the United States once the H-1B has been secured—they are not faced with the threat of removal from their country of residence if the H-1B application is not selected. As Mohit, who holds a Bachelor's degree in computer engineering from an Indian university, noted, “I applied for my H-1B visa from [India]… I didn't get it, so I had to wait for a year, and then I applied again in 2010. And then I got it. That was it. It wasn't a huge deal.”

Once respondents obtained their visas, their frustrations shifted from the uncertainty around winning the lottery to the rigidity and constraints of the visa. Despite the H-1B Portability Act of 2000, which allows H-1B employees to transfer their visa to a new employer sponsor, many respondents said they felt tied to their employer. Both student migrants and direct recruits voiced a sense of powerlessness to move between jobs because they saw their visas as tied directly to their employer, and felt they lacked negotiating power. Direct recruits in the sample also experienced constraints in their job mobility because of smaller professional networks in the United States, a devalorization of their degrees, and occasional threats from their employers.

When Ridhi, who first arrived as an international student, became frustrated with her manager's “unreasonable expectations” and “bad leadership style,” she said she felt “trapped” and unable to quit, because she believed she would have to leave the country if she did so.

I can't just get up and quit if work gets frustrating. I knew a couple of people that I worked with—they just up and quit. They just didn't show up the next morning. But I can't do that, because I would have to go back home and pretty much put my life on hold. Leaving the company would mean leaving the country.

Others echoed this frustration about their entwined legal status and employment status. “I can't be jobless in the U.S… If I'm not working, I can't be in the country,” said Vikram, who earned his Bachelor's degree on the West Coast and works as a tech consultant.

Ridhi also worried about losing her job and the implications it would have for her legal status.

You live in fear of a bad performance review. The way the H-1B works is that if you do get fired, then you have to leave that very day. That was not a position I wanted to be in. It's definitely a cause for worry. It's pretty stressful.

Other H-1B migrants who first arrived as international students felt that the visa constrained their ability to look for job opportunities with new employers. They felt nervous about losing their current job if their employer found out that they were exploring other options, which would result in falling out of status. Mira has been working at the same company since she graduated from college on the East Coast four years ago. She said she would like to explore opportunities elsewhere but is nervous about doing so because of the risks it poses to her employment.

I don't feel like I can switch employers. It's tricky, there's too much risk. I don't want them to know I'm looking somewhere else, because then I could end up with no job at all… they could fire me.

Even those who found a new job often thought twice before leaving their employer, because they were anxious about the transfer of paperwork, and the potential challenges that could arise from transferring their visa. Varad, who went to college in the United States and transferred his H-1B to a new employer last year, noted that this transition was nerve-wracking. “When I was changing jobs, I had this thing in the back of my mind of what's going to happen to me if something got wrong with my visa transfer or something? It was a pretty big risk.” Because H-1B migrants feel tied to an employer, they see their ability to negotiate and leverage competing offers in the U.S. labor market as conscribed. Varad said this imbalance frustrates him^8^.

I don't have any negotiating power, because I'm at the will of the company. I can't leverage other offers to get a promotion, or to get them to increase my salary. If I were a permanent resident, I could shop around, test the waters. But I can't, I have no leverage with my employer. I need them more than they need me.

Indian-educated respondents voiced similar frustrations. Amit, who has a Bachelor's degree in software engineering from India, has worked as a subcontracting consultant for same oil company in Houston since he moved to the United States on an H-1B 4 years ago. He feels that his visa makes it “almost impossible” to switch employers. “It's a big stinking pile of mess…. You're basically trapped. I was disappointed…you start regretting…why would I move here in the first place?”

For some H-1B direct recruits, company practices created additional barriers to job mobility. When they did manage to find a new job, some faced active resistance and threats from employers. Employers threatened to withhold work authorization paperwork or asked workers to pay exit fees when workers informed them of their resignation. When Roshan gave his 2 weeks notice at his subcontracting company, “they weren't too happy about it…I had trouble getting my paperwork transferred… it took a while, it was pretty stressful.” When Satya, who moved to the United States with an Indian-based subcontracting company, told his first employer that he was leaving, his employer asked him to pay an exit fee and delayed his paperwork transfer for 2 years^9^.

My contracting company asked me for $10,000… to give my papers back. [They] said you're not allowed to leave the company…I didn't pay them…it took two years to get my papers. It's an unwritten rule. Most of the contracting companies do that… they ask you for a lot of money or they hold the papers…three of my friends never got their papers.

Direct recruits were further constrained in seeking new jobs because of their smaller professional networks in the United States. While some had friends and family in the United States, many described their professional networks as rooted to their current workplace. This made it challenging for some like Mohit to find opportunities elsewhere. “It would be nice to work somewhere else, but I don't really know where to start…who to ask. Most of the people I know here are from work, so I can't really talk to them about it,” he said. He eventually used LinkedIn to expand his professional network but has been unable to find a new job and is still working for his original employer.

In contrast, respondents who studied in the United States described much larger networks that provided support and information about jobs across the country, even if they felt constrained in their job mobility in other ways. “A ton of my friends from my Master's program stayed in the U.S.,” said Abhinav, who studied engineering on the East Coast. “We don't see each other much, because they live all over the country, but we have group chats and I call them when I want to complain about work… A few of them work with me, for the same company…we helped each other get jobs.”

International student respondents often oriented their reference group to native-born peers and expressed high levels of frustration with the constrained job mobility on the H-1B. Amala, for example, said that she gets frustrated when she talks to her native-born friends, who she met during her Bachelor's studies in the United States, about their career prospects. “There's just so much more for me to think about before I make a career move, [my American friends] don't even realize it,” she said. “I could be in serious trouble if I lost my job… because of my visa. I don't think it's fair really. We all went to college together, my GPA is higher than theirs…but because I'm on an H-1B, none of that matters… it's really annoying.” Mira echoed similar frustrations about her native-born friends' job search, which she perceived as much “easier” than her own. “It was so hard, so time consuming, so exhausting for me to find a job… because of my visa needs. My friends had no idea how good they had it. They didn't need to jump through these H-1B hoops.”

Similarly, Rupi made sense of her job mobility experiences in reference to her native-born peers. She describes the disparities as frustrating and unfair. “Nobody likes looking for jobs, I know that,” said Rupi. “I think it makes it an uneven playing field, people who don't have to worry about their visas. Americans are more confident, they have a better chance of…getting the [job].” Vikram echoed these sentiments. “American are always going to have something I can't have: peace of mind.”

Though some direct recruits faced threats from their employer and stringent working conditions, they still expressed relatively high levels of satisfaction. Maintaining life in India as their point of reference, they compared their work experience in the United States to their work experience back home. Myan said that he was pleasantly surprised by the work-life balance at his current job as a computer programmer in Ohio. After graduating with a Bachelor's degree from a top university in India, he was working in Mangalore for a software engineering company, and was hesitant to move to the United States because his father had chronic health issues. But once he transferred to the United States through his company, he realized that things were “better here…you can have a really good work-life balance here. The weekends are completely your own. When I was working in India, I had to go work on the weekends and work late into the night…It's not really comfortable for any human being.” Though he is still on-call one Saturday a month, Myan says the work conditions are better now that he is in the United States.

Roshan, who also moved directly on an H-1B visa, echoed these feelings. “One thing I like about living here is just in terms of work-life balance,” he said as he described his work at a consulting firm in New York. “It's a lot better in the U.S. In India, the work hours are a lot longer, commutes are longer, so work-life balance suffers a lot.” Even Satya, who described his first employer as “authoritarian,” said that he was happier with his lifestyle in the U.S than he was back home. “There were 11 of us sharing a two-bedroom apartment for almost a year [in India]. It was super crazy – no bathroom time, that kind of thing. Now I live in… an apartment with lots of space. That's what guided me …when things were tough. Whatever I can't do in India, here I can do it.”

Respondents' reference groups also influenced their settlement intentions. For student visa migrants, the desire to settle permanently in the United States that initiated their migration was deepened by their tight social ties to U.S. citizens and people with permanent status in the United States. “When we were graduating, everyone was staying here, so I figured I would stay here too,” said Vikram.

The degree of acculturation, the density of social networks in the United States, and intensity of permanent settlement intention were powerful forces contributing to feelings of anxiety and alienation among respondents. Despite having the deepest ties to life in the United States, respondents who wanted to settle permanently felt the least secure and most alienated by the temporary nature of the H-1B visa. They felt stressed about the uncertainty of the 6-year limit, and often expressed resentment about the constraints and insecurity of their legal status. These feelings were most acute among those who felt they had the most to lose—migrants who planned to settle permanently and felt rooted in the United States. Respondents with permanent settlement intentions, mostly international students-turned skilled migrants, expressed feelings of liminality that were in tension with their feelings of belonging and permanence in the United States. Those who planned to emigrate, mostly direct recruits, also expressed feelings of liminality, yet these feelings resonated with their temporary sense of migration and ultimate plans to return home. For respondents who planned to settle permanently in the United States, entwined employment and immigration statuses were a source of anxiety; for those who planned to leave, the entwined statuses were a source of frustration and a reason to emigrate. Dev, who earned his JD in the United States and is in the process of applying for a green card through his employer, noted the tension between his feelings of belonging and his feelings of impermanence.

We're getting into that green card picture right now. It really did feel like a massive, massive relief. To an astonishing degree. I just realized, wow I've really been carrying a lot of background stress about this for a while. I feel like this place is totally my home… I love it… so it's almost weird that there's this fundamental…legal impediment to that really being an accurate description of things.

Mira also described feelings of belonging in the United States, and concern that a change in her visa status could disrupt her plans to stay.

Why would I leave? My life is here. My work, my friends, my boyfriend… they're here. I've spent my entire adult life in this country, this is the only place I've ever lived on my own… I didn't grow up here, but all of the important things in my life have happened here… I feel American, but my passport is Indian…there's always that worry that something could change and I would have to leave.

Migrants who saw their migration as temporary, mostly direct recruits, also expressed a sense of liminality due to the intersecting employment and legal status, but the temporary nature of the visa resonated with their temporary plans. Alok, who holds an Indian degree and works in computer programming, describes Ridhi's “worst-case scenario” of losing his job and needing to leave the country in lighter terms. “I actually lost my job at one point, and had to go back home for a while to figure out my next move, because I couldn't be in the U.S. It wasn't legal. So I had to leave the U.S. and come back with a new stamp…that was about it. It was kind of annoying, but I was going to move home eventually, so it didn't really matter.” He describes this experience as an annoyance, rather than a “major life disruption,” as Ridhi did.

Respondents who did not intend to settle permanently engaged in short-term decision making, which reinforced their likelihood to move home. This sense of liminality rooted in their legal and employment status lead migrants to delay basic settlement behavior. “I waited two years to buy furniture for my apartment because I wasn't sure how long I would be here… if I would have any trouble with the H1,” said Jai. Myan, a direct H-1B recruit, expressed a similar logic in explaining why he rents his apartment month to month. “I'm not going to be here forever… and you never know if something's going to happen with your visa… I didn't even sign a one-year lease, what if I have to leave in the middle of it?”

### The Settlement/Emigration Decision: Opting Out of the Skilled Migration System

Many respondents ultimately opted out of the skilled migration system, either by returning home to India, or finding other pathways to permanent residence in the United States. Only a small portion of respondents indicated plans to pursue an employer-sponsored green card, mostly direct recruits without career prospects back home or strong social ties to the United States. Where many direct recruits opted out of the system entirely and moved home to pursue career opportunities there, many student visa migrants found alternative pathways to obtaining permanent residence through family reunification programs, either getting sponsored by a family member living in the United States or marrying a U.S. citizen to get a green card.

Some respondents decided to move back to their country of origin before their H-1B expired, frustrated by the employment restrictions imposed by the visa. Parth went to college in India and moved back to his hometown of New Delhi after working in the United States for 4 years because he wanted to start his own company, which is not authorized on the H-1B visa.

I want to travel and I want time to do my own thing, to work on my own projects. I couldn't really do that in the U.S. on my [H-1B]. If I could have stayed on in the US and done my own thing there and not had a job for a while I would have considered that. But that was not an option.

For both Parth and Alok, who also moved back to his hometown of Delhi, the employment possibilities in India outweighed the restrictions of staying in the United States on an H-1B visa. Alok was excited about the flexibility of career options available to him at home.

I wanted to go back and recalibrate what things were on the ground were like in India, professionally, in terms of work culture and opportunities and stuff. I was trying to figure out types of opportunities, what I could be doing in India. I don't have the restrictions I had in the U.S., because I'm a citizen here. That was a nice change.

Parth similarly saw new opportunities at home.

Work is definitely way more exciting over here. There's so much opportunity to do your own thing. That's the really rewarding part of it. That's why I moved back, I wanted to have my own business. That was definitely a goal.

For respondents who did want to stay in the United States, they often came up against the complexities of the green card process. Where student visa migrants found alternative pathways to green cards, direct recruits cited the complicated nature of the employer-sponsored green card as a key reason for emigration. Like the H-1B, the employer-sponsored green card is granted at the discretion of the employer, and some companies rarely sponsor workers. Myan, a direct recruit, said his subcontracting company rarely sponsored workers for a green card, and so he planned to move home when his H-1B expired. “I haven't heard of anyone at my company getting sponsored for a green card. It's just not something they do. Most people [at my company] don't even stay the full six years, we kind of come and go. They want to get fresh talent.” The problems Satya described with his paperwork transfer also contribute to the small number of green cards conferred by outsourcing companies.

Some respondents did work for employers willing to sponsor their green card, but the duration of the green card process was a deterrent. Once the requisite 2-year processing period is over, a worker does not automatically obtain a green card. They then receive a priority date, which adds them to the queue of prospective immigrants in their country of origin—it could take up to 10 years for an Indian national to officially apply for the green card. Amit cited the long wait time and the uncertainty related to it, as the primary reason for not pursuing permanent residence in the United States.

No, I'm not going to apply for a green card. That would sound good, but given the wait time, I'm really discouraged. The number of years that it takes for a green card to be processed is honestly outrageous. [With] all of the insecurities of my job and everything else…honestly, it's like a sword hanging on top of your head.

Frustrations with the migration process are not the only factor at play in the emigration decision—personal preferences for life in India and living closer to family also contributed to respondents' emigration decision. Some always planned to move home and saw working in the United States as an opportunity to gain skills while paying off student loans incurred while gaining United States degrees. But for this group of migrants in high demand on the global labor market, it is important to understand how the visas that enable them to work here might play a role in shaping their desire to leave.

While most migrants in this study had not finished the 6 years of their H-1B visa, many who did plan on settling permanently in the United States had begun the process of making alternative arrangements for permanent residency, mostly through family reunification programs. Some planned to get married to obtain a green card, while others sought sponsorship through a sibling or cousin.

Nisha, who expressed a strong desire to become a U.S. permanent resident, said that she and her long-time boyfriend, a U.S. citizen, recently got engaged. “I'm getting close to the end of my H-1B and I realized it was going to be a huge headache to get a green card through work. I didn't want to spend ten years working at the same company, waiting to see what would happen. I just wanted to start my life here already. So [my boyfriend] and I decided the best thing to do would be to get married. It probably would have happened eventually anyway, but this definitely sped up the timeline.” Abhinav also planned to obtain a green card through a family member. “I really like living here, and I don't want to leave. This feels like my home now. But my visa's going to expire eventually, so I'm looking into alternative arrangements. I have a cousin who's American, and we're trying to figure out if he can sponsor me. If that doesn't work out, I'm not sure what I'll do,” he said.

Student migrants who planned to settle permanently expressed a familiarity and comfort with undergoing legal status transitions, having already successfully moved from an F-1 visa to an H-1B. Vikram said learning about the green card was much less intimidating after navigating the H-1B system. He knew where to get information, and described the process as less complex, relative to the H-1B. He planned to get a green card through family reunification channels rather than employer sponsorship, which he said sounded as complicated as the H-1B process. “I started looking into ways to get a green card a few years ago, just to know what my options were. Honestly, it's way better than the H-1B mess, I mean, if you try to get it through your job, that sounds like a nightmare, but I'm just going to apply through my cousins and it should be done in a year or so. No big deal.”

In contrast, most respondents who did plan to pursue the path of employer-sponsored green cards had little prior experience with transitioning between legal statuses. Most had obtained their H-1B through an employer as direct recruits and had less experience navigating the U.S. immigration system. Respondents also described a general confusion and lack of information and resources in navigating the process of obtaining an employer-sponsored green card. For respondents pursuing LPR status, most expressed an interest in obtaining “a green card,” but did not differentiate between the various categories of green cards. Most interviewees were clear on the difference between green cards obtained through family reunification channels and employer-based green cards, but respondents were often surprised by details of the application process, like the long wait times, and were not familiar with the differences between the three primary employer-based green cards, EB-1, EB-2, and EB-3, which confer different levels of priority to applicants based on skill level (USCIS, 2019).

Satya, despite having many complications with paperwork transfers from a previous employer, decided to apply for a green card through his current employer, and they have begun to file the paperwork. He described the beginning of the application process as a steep learning curve, in which he had to do a lot of outside research on his own. “My company's sponsoring me, but they aren't explaining much about how the process works. I'm learning this as I go… I've been reading a lot of the online forums about the green card process, because I don't know how it works or what to expect.” When asked which green card he was applying for, he said he had to check and paused the interview for a few minutes while he searched in his email before confirming that he was applying for an EB-3 visa

Because he maintained India as his reference group, he is more patient about the complicated wait times. “I don't mind waiting a long time for the green card. I have it pretty good, I like my job, and I think it's all going to work out. Even when my last employer wouldn't transfer my documents, I figured it out in the end… it's worth it for staying here.”

## Discussion

Taking a life-course perspective on skilled migration, this paper illuminates the micro-level processes and various pathways that lead to permanent settlement and emigration, and identifies legal status transitions as a key sorting mechanism in immigrant selection. By tracing the different channels that migrants follow to obtain a skilled work visa, with an eye toward eventual permanent residency, I unpack the micro-level selection effects at play in the process of skilled migration. I examine whether a skilled migrant pursues a green card, and whether they pursue it through channels of skilled migration or family reunification. Taking a long view of the migration journey across multiple legal status transitions, this paper reveals new selection mechanisms not discussed in prior research.

This paper unpacks the specific factors that lead to selection out of the skilled migrant program in the transition from a student visa to a temporary work visa to an employer-sponsored green card. Migrants who successfully underwent a prior legal status transition were more likely to pursue permanent residence, but also saw a wider array of avenues to obtain a green card. Expanding on Jasso et al.'s (2005) notion of “visa stress,” I find that the mismatch in some migrants' permanent settlement intentions and temporary legal status can lead to feelings of alienation and frustration in the immigration system and the U.S. labor market, driving some to seek channels outside of skilled migration to obtain a green card. Other migrants with weaker social ties and less institutional attachment to the U.S. felt less of a disconnect between their expectations and settlement opportunities, and thus were more willing to pursue employer-based green cards. A third group, with exciting job prospects at home or abroad, dropped out of the system entirely and decided to emigrate.

By focusing on the pathways in and out of permanence, the findings of this paper offer rich insights into the tensions and unintended consequences of immigration law. One example is the disconnect between the settlement intentions expressed among F-1 visa and H-1B visa migrants, and the provisions of U.S. immigration law. Many respondents in this study who arrived on an F-1 student visa expressed an interest in settling permanently in the United States, despite the fact that F-1 visa regulations mandate that applicants indicate non-immigrant intent on their visa applications and demonstrate an “intention to depart the United States upon completion of the course of study” (Batalova, 2006; U.S. Department of State, 2019). In contrast, migrants in this study who arrived on an H-1B visa often expressed an ambivalence about permanent settlement, even though the “dual intent” H-1B visa allows migrants to eventually apply for permanent residence (Jasso, 2010; Sahoo et al., 2010). The findings of this study are consistent with Jasso's (2009) findings that visa holders are less likely to express settlement intentions than other immigrants, and with Jasso et al.'s (2010) finding that the primary pathway to permanence for migrants who arrived on F-1 visas is to obtain a green card through marriage to a U.S. citizen, though they challenge Lowell (2005) and Batalova's (2006) findings that about half of H-1B migrants eventually apply for LPR status.

The disconnect between stated settlement intentions, observed settlement behavior and the provisions of U.S. immigration law is a rich site for further study and has both social scientific and policy implications. It illuminates the influence and limitations of immigration policy in regulating migration flows and the driving forces leading to unintended consequences in immigration policies (Massey, 2013). Our understanding of these unintended consequences would be enriched by further study of the gap between immigrant intentions and behavior among F-1 and H-1B migrants, which could indicate whether migrants are using certain visa pathways with intentions that conflict with the provisions of the visa. Further, this disconnect reveals the drop-out points in the skilled migration process, leading to selection of certain characteristics among skilled migrants. Immigration researchers will see the importance of understanding how and why migrants drop out of legal systems to develop stronger models of macro-level selection effects and better understand the unintended consequences of immigration policies.

The findings of this paper also highlight the need for more robust and granular longitudinal data on legal status^10^. Because of inadequate and incomplete administrative data, current measures of observed settlement behavior through adjustment of a temporary to a permanent status are often limited and inconclusive (Batalova, 2006). More robust data on legal status would allow for systematic comparisons within and between legal status categories and for the construction of migration history data to examine legal status trajectories, pathways and drop-out patterns. These findings emphasize the importance of specificity in studying visa categories and in identifying variation in the migration histories and ascribed characteristics of migrants within a single visa group.

Policymakers interested in understanding the composition and outcomes of various classes of admission to the United States will benefit from the findings of this paper as well. Who is arriving, and under what visa? How long do they stay? How does visa policy design play out on the ground? As Massey (2013) notes, immigration policies often do not produce the desired results, and in many cases can produce unintended consequences. The feelings of liminality expressed by some respondents suggests that a “probationary” admission system based on temporary visas may ultimately delay or redirect final integration outcomes. Further, the reasons that some migrants in this study cited for emigrating suggest that current policies are losing out on opportunities to recruit and retain migrants who develop skills in the United States. In addition, the complexities of legal status transitions, and the specific opportunities and constraints enabled by various visa categories, should be more clearly and readily communicated to migrants.

Finally, this paper weighs in on a long-standing theoretical debate about the power and efficacy of the state in regulating immigration policy. The U.S. government is experiencing a partial undoing of its bordering capacity. This paper illustrates the ways that various migrant groups navigate migration systems regardless of policy design. At the same time, the U.S. government is engaging in neoliberal immigration policies which place increasing power in the hands of private corporations, who wield control over immigrants' legal statuses through their employment status. And this power is concentrated in the hands of fewer and fewer companies, as a shrinking pool of corporations dominate the H-1B migration system (USCIS, 2018).

## Ethics Statement

This study was carried out in accordance with the recommendations of the Institutional Review Board and the protocol was approved by the University of Pennsylvania IRB review board.

## Author Contributions

EJ contributed conception, design, data collection and analysis for the study, and wrote the manuscript.

### Conflict of Interest Statement

The author declares that the research was conducted in the absence of any commercial or financial relationships that could be construed as a potential conflict of interest.

## Footnotes

^1^This 2016 paper in *International Migration* sheds light onto many of the major dynamics involved in return migration for Indian migrants. Where the Sabharwal and Varma paper examines decision making at the intersection of economics and psychology, I focus on the broader social dynamics at play.

^2^The emphasis in previous work on unskilled and undocumented migrants makes it hard to separate the effects of a temporary legal status from the multitude of other challenges that vulnerable low-wage immigrant workers face. By focusing on a relatively privileged group of migrants with high levels of education working in white-collar professions, I aim to disentangle some of these effects and zero in on the role of a temporary legal status. This approach follows in the tradition of Gonzales's (2016) work, which shows how legal status can block incorporation for undocumented migrants with high levels of education. Further, many temporary migrants fall into racialized groups; Golash-Boza and Hondagneu-Sotelo (2013), Ngai (2004) and others have expertly illustrated the key role that race plays in the immigrant incorporation. While a systematic analysis of the racialized experience of skilled migration is beyond the scope of this paper, see (Banerjee, 2010)'s work for an in-depth exploration of how Indian migrants working in IT sectors experience race in the workplace. My goal here is not to create a false equivalence between H-1B migrants and other legal status groups facing many deeply concerning dimensions of legal violence, but rather to focus on how employers leverage temporary legal statuses as another mechanism of exploitation and control at all levels of education and occupation.

^3^There are five categories of employer-sponsored green cards, which are ranked in order of preference according to a migrant's skill level and classification (USCIS, 2019). EB-1 visa holders include professors and multinational executives; EB-2 visas are for migrants with exceptional abilities; EB-3 holders include skilled workers and professionals for which qualified workers are not available in the United States. EB-4 visas, less common, are reserved for religious workers, translators and certain doctors, broadasters and military personnel; EB-5 visas are often called investor visas because they are reserved for migrants who invest a minimum of $500,000 into the U.S. economy and create jobs for U.S. workers. See Lowell (2001) or Jasso et al. (2010) for a more detailed description the EB visa categories, and North (2012) for a specific discussion of the EB-5 investor visa.

^4^Because of the sample design of this study, most interview respondents in this paper fall into the EB-3 category. To transition to an employer-sponsored work visa, H-1B visa holders need to obtain sponsorship from their employer. F-1 visa holders do not have a direct path to employer-sponsored LPR status, and must either first transition to an H-1B or other work visa, or obtain LPR status through family reunification channels (Jasso et al., 2010). As discussed in the findings section, respondents in this study who initially arrived on F-1 student visas often sought LPR status through means of family reunification.

^5^While these studies sensitize us to some of the patterns and dynamics at play in the transition from a temporary to permanent legal status, because of inadequate and incomplete administrative data and the specificity of U.S. immigration provisions and visa classifications, measures of observed settlement behavior through the adjustment of a temporary to a permanent status are often limited and inconclusive (Batalova, 2006). Other studies, such as analyses of the New Immigrant Survey, offer useful insights but are based on a survey of green card holders, rather than a representative sample of F-1 and H-1B visa holders.

^6^For respondents with mixed educational backgrounds, such as those who have a Bachelor's from an Indian University and an Master's from a U.S. university, these dynamics might play out slightly differently, but for the purposes of this paper, I explore the effects of all U.S. educational backgrounds together.

^7^I interviewed five additional respondents who were not approved for the H-1B. For analytical purposes, they are not included in this sample, but most emphasized high levels of anxiety while they waited for the H-1B lottery results, which they sometimes applied to multiple years in a row. Some enrolled in master's programs to maintain legal status; others moved home for a year with the intention of applying again; others got married to pursue legal status through other channels.

^8^There might be some benefits to staying at a single company for an extended period of time — workers can be rewarded with mentorship and promotions. This model mirrors traditional career structures where workers advance up an internal career ladder (Kalleberg and Sorensen, 1979). However, the overarching career mobility structure in the tech industry is to job hop, to advance through diagonal moves between different companies to gain experience and increase earnings (Fallick et al., 2006; Freedman, 2008).

^9^Work certification paperwork is essential in applying for a green card, so withholding work experience papers would render an H-1B migrant unable to permanently settle in the United States on an employer-sponsored green card.

^10^Recent debates about the inclusion of a citizenship question on the 2020 U.S. Census speak directly to this issue. This question has been weaponized as an anti-immigrant attempt at depressing Census participation in immigrant communities, which could result in less Congressional representation in those areas. While previous studies suggest that the inclusion of legal status questions does not necessarily lead to lower response rates (Bachmeier et al., 2014), the heightened political climate in which this question is being introduced raises serious concerns about the intent and application of this question on the 2020 Census. Until impacted communities are more at ease that data on legal status collected by the Census Bureau will not be shared with other U.S. agencies like Immigration and Customs Enforcement, surveys on legal status should be conducted by independent organizations and administered in settings that emphasize data security and community trust. Further, more targeted surveys on legal status will allow for more granularity in the detail of questions about legal status across the life course.
